# Supplementary material for: Algal PETC-Pro171-Leu suppresses electron transfer in cytochrome b6f under acidic lumenal conditions
Source: Plant Physiol. 2022 Dec 14;191(3):1803–17. doi: 10.1093/plphys/kiac575 (PMC10022631; doi:10.1093/plphys/kiac575)
Supplement: kiac575_Supplementary_Data [file kiac575_supplementary_data.pdf]

Supplemental Figure S1

```
                     ....|....| ....|....| ....|....| ....|....| ....|....|
C. reinhardtii       ---------- ---------- -MAMLSSRRV AAPAKASAIR RSR----VMP  25
A. thaliana          MASSSLSPAT --QLGSSRSA LMAMSSGLFV KPTKMNHQMV RKEKIGLRIS  48
S. oleracea          MASFTLSSAT PSQLCSSKNG MFAPSLAL-A KAGRVNVLIS KERIRGMKLT  49
M. laminosus         ---------- ---------- ---------- ---------- ----------  1
Nostoc sp.PCC7120    ---------- ---------- ---------- ---------- ----------  1
S. cerevisiae        ---------- ---MLGIRSS VKTCFKPMSL TSKRLISQSL LASKSTYRTP  37
Clustal Consensus

                     ....|....| ....|....| ....|....| ....|....| ....|....|
C. reinhardtii       VVRAAAASSE VPDMNKRNIM NLILAGGAGL PITTLALGYG AFFVPPSSGG  75
A. thaliana          CQASSIPADR VPDMEKRKTL NLLLLGALSL PTGYMLVPYA TFFVPPGTGG  98
S. oleracea          CQATSIPADN VPDMQKRETL NLLLLGALSL PTGYMLLPYA SFFVPPGGGA  99
M. laminosus         -MAQFTESMD VPDMGRRQFM NLLAFGTVTG VALGALYPLV KYFIPPSGGA  49
Nostoc sp.PCC7120    -MAQFSESVD VPDMGRRQFM NLLTFGTVTG VALGALYPVV NYFIPPAAGG  49
S. cerevisiae        NFDDVLKENN DADKGRS--Y AYFMVGAMGL LSSAGAKSTV ETFISSMTAT  85
Clustal Consensus                 .*  :       :  *                *:..  .

                     ....|....| ....|....| ....|....| ....|....| ....|....|
C. reinhardtii       GGGGQAAKDA L-------GN DIKAGEWLKT HLAGDRSLSQ GLKGDPTYLI  118
A. thaliana          GGGGTPAKDA L-------GN DVVAAEWLKT HGPGDRTLTQ GLKGDPTYLV  141
S. oleracea          GTGGTIAKDA L-------GN DVIAAEWLKT HAPGDRTLTQ GLKGDPTYLV  142
M. laminosus         VGGGTTAKDK L-------GN NVKVSKFLES HNAGDRVLVQ GLKGDPTYIV  92
Nostoc sp.PCC7120    AGGGTTAKDE L-------GN DVSVSKFLES HNVGDRTLVQ GLKGDPTYIV  92
S. cerevisiae        ADVLAMAKVE VNLAAIPLGK NVVVKWQGKP VFIRHRTPHE IQEANSVDMS  135
Clustal Consensus          **    :       *: :: .    :.      .*   :   :.:.. :

                     ....|....| ....|....| ....|....| ....|....| ....|....|
C. reinhardtii       VTADSTIEKY GLN------- -AVCTHLGCV VPWVAAEN-K FKCPCHGSQY  159
A. thaliana          VENDKTLATY GIN------- -AVCTHLGCV VPWNKAEN-K FLCPCHGSQY  182
S. oleracea          VESDKTLATF GIN------- -AVCTHLGCV VPFNAAEN-K FICPCHGSQY  183
M. laminosus         VESKEAIRDY GIN------- -AVCTHLGCV VPWNAAEN-K FKCPCHGSQY  133
Nostoc sp.PCC7120    VESKEAITDY GIN------- -AVCTHLGCV VPWNAAEN-K FKCPCHGSQY  133
S. cerevisiae        ALKDPQTDAD RVKDPQWLIM LGICTHLGCV PIGEAGDFGG WFCPCHGSHY  185
Clustal Consensus    .  .        ::         .:*******      .:    : ******:*

                     ....|....| ....|....| ....|....| ....|....| ....|...
C. reinhardtii       NAEGKVVRGP APLSLALAHC DVAESGLVTF STWTETDFRT GLEPWWA- 206
A. thaliana          NAQGRVVRGP APLSLALAHA DIDEAGKVLF VPWVETDFRT GDAPWWS- 229
S. oleracea          NNQGRVVRGP APLSLALAHC DVDD-GKVVF VPWTETDFRT GEAPWWSA 230
M. laminosus         DETGKVIRGP APLSLALCHA TVQD-DNIVL TPWTETDFRT GEKPWWV- 179
Nostoc sp.PCC7120    DATGKVVRGP APKSLALSHA KTEN-DKIVL TSWTETDFRT GEEPWWS- 179
S. cerevisiae        DISGRIRKGP APLNLEIPAY EFDG-DKVIV G--------- -------- 215
Clustal Consensus    :  *:: :** ** .* :          . : .
```

The amino acid sequence alignment for Rieske ISP

The amino acid sequences of PETC protein in five photosynthetic organisms and of Rieske protein of cytochrome bc_1 complex in yeast are aligned: *Chlamydomonas reinhardtii* (C. reinhardtii), *Arabidopsis thaliana* (A. thaliana), *Spinacia oleracea* (S. oleracea), *Mastigocladus laminosusand* (M. laminosus), *Nostoc* sp. PCC 7120, and *Saccharomyces cerevisiae* (S. cerevisiae). Clustal consensus are shown as following: conserved (asterisk), strong similarity (semicolon), and weak similarity (comma). The numbers on the right show the position of the amino acid. The chloroplast targeting transit peptide is marked with black colored rectangle, the hinge region is marked with green rectangle (Yan and Cramer, 2003), the four amino acids for ligands of iron is Rieske ISC are marked with blue colored rectangles, and the proline residue substituted by leucine in this study is marked with magenta colored rectangle.

The amino acid residues mutated in *bc*$_1$ complex (marked in orange-colored rectangles) resulted in slower *b*-heme reduction. The transit peptide is determined experimentally for *S. oleracea* (Pfefferkorn and Meyer, 1986) and *C. reinhardtii* (Pierre et al., 1995) but is predicted by ChloroP 1.1 (chloroplast targeting is predicted and a 50 amino acid is estimated for transit peptide with 0.552 total Score and 4.079 CS-Score) for *A. thaliana*. Amino acid sequences are from Cre11.g467689 (JGI version 5.6) for *C. reinhardtii*, AT4G03280.1 (TAIR) for *A. thaliana*, P08980 (UniProt) for *S. oleracea*, P83794 (UniProt) for *M. laminosusand*, Q93SX0 (UniProt) for *Nostoc* sp. PCC 7120, and P08067 (UniProt) for *S. cerevisiae*. The four amino acids for ligands of two irons in a Rieske ISC (Cys134, His136, Cys152, His155 in *C. reinhardtii*; Cys157, His159, Cys175, His178 in *A. thaliana*; Cys158, His160, Cys176, His179 in *S. oleracea*; Cys108, His110, Cys126, His129 in *M. laminosus* and *Nostoc* sp. PCC7120; Cys159, His161, Cys178, His181 in *S. cerevisiae*) and flexible hinge linker (from Ser72 to Gly79 in *C. reinhardtii*, from Gly95 to Gly102 in *A. thaliana*; from Gly96 to Gly103 in *S. oleracea*; from Ser46 to Gly53 in *M. laminosus*; Ala46 to Gly53 in *Nostoc* sp. PCC7120; from Thr85 to Ala92 in *S. cerevisiae*) are conserved. As well as these amino acids, the mutated Proline residue in Arabidopsis *pgr1* (Pro171 in *C. reinhardtii*; Pro194 in *A. thaliana*; Pro195 in *S. oleracea*; Pro145 in *M. laminosus* and *Nostoc* sp. PCC7120; Pro197 in *S. cerevisiae*) is conserved in amino acid sequence alignment.

Supplemental Figure S2

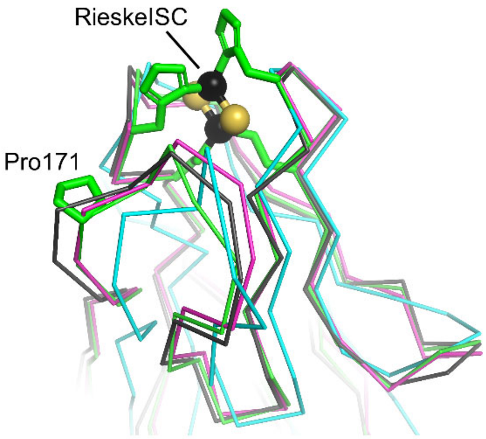

The PETC protein conformation around Rieske ISC and the proline residue mutated in this study. The four b_6f structures (PDB IDs and color code are; 1Q90 with green for *C. reinhardtii*, 1VF5 with cyan for *M. laminosus*, 4OGQ with magenta for *Nostoc* sp.PCC7120, and 6RQF with gray for *S. oleracea*) are overlayed with the secondary structure between Cys134 to His155 in *C. reinhardtii* of which side chains are ligands for Rieske ISC. A ball-stick model shows the Rieske ISC in *C. reinhardtii* and the side chains involved in ligation, as well as proline 171.

Supplemental Figure S3

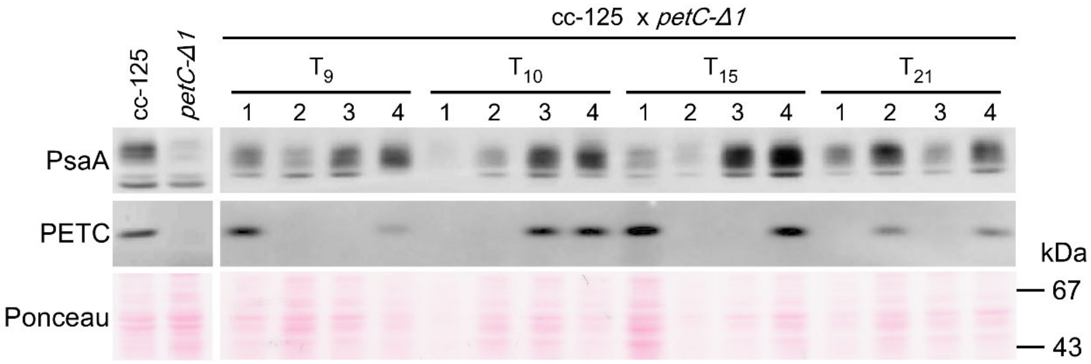

Isolation of Photosystem I (PSI) protein reduction free PETC deleted strain

The *petC-Δ1* was backcrossed with cc-125 and the resultant progenies were analyzed by immunoblotting using the antibodies against PsaA or PETC. Whole cellular proteins (0.5 µg Chl per lane) were denatured, separated by SDS-PAGE, and analyzed by immunoblot. The polypeptides transferred on nitrocellulose membrane were stained with Ponceau (the migration positions of protein standard are shown). The proteins of progenies (1 to 4) from four independent perfect tetrads (T_9, T_{10}, T_{15}, and T_{21}) and their parent strains (cc-125 and *petC-Δ1*) are loaded.

Supplemental Figure S4

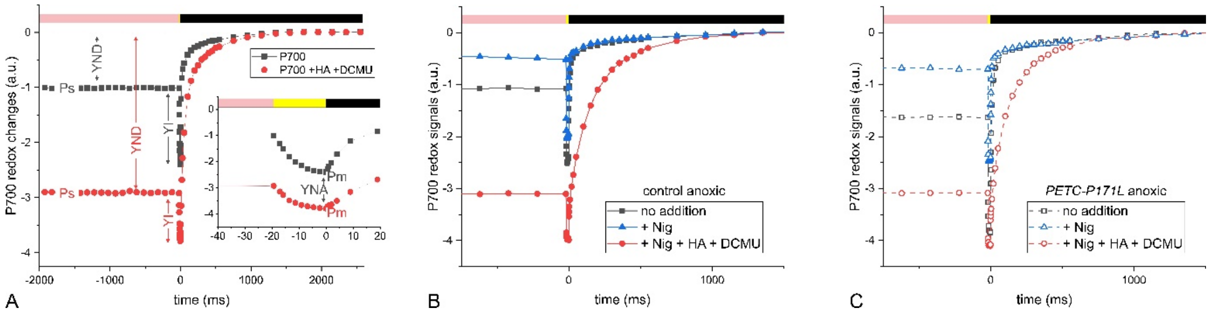

P700 measurements are shown. (A) Representative steady-state P700 redox signals (Ps) were recorded in an oxic control strain after adaptation to actinic background light (pink bar), followed by a 22-ms saturating pulse (yellow bar, insert). The pulse yielded maximal $P700^{+}$ (Pm, insert). The latter was further increased in the absence of Photosystem II (PSII) activity (red circles, + hydroxylamine (HA) + 3-(3,4-dichlorophenyl)-1,1-dimethylurea (DCMU)) due to elimination of acceptor side limitation (YNA). Pre-oxidized P700 in the light was due to donor side limitation (YND) and the photo-oxidizable P700 population during the pulse represented the yield of P700 (YI). **(B)** Control cells and **(C)** *PETC-P171L* cells under anoxic conditions. Nigericin (Nig) and PSII inhibitors (HA and DCMU) were present when indicated. The *PETC-P171L* cells showed larger YND and YI (black squares). Nig addition diminished YND and PSII-inhibited cells showed comparable kinetics in both samples.

Supplemental Figure S5

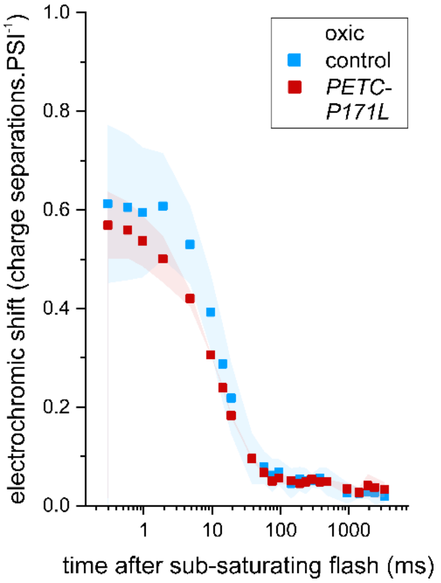

The electrochromic shift signals following a sub-saturating laser flash are shown in oxic conditions. The ~10-ms rising phase, associated with charge separation activity upon a single b_6f turnover, was masked by fast ATP synthesis rates that caused a signal decay within ~50-ms (cf. Figure 3E in the main text for anoxic conditions). ATP synthesis was slightly faster in oxic light-adapted *PETC-P171L*. Sub-saturating flashes were fired on light-adapted cells after a 30-s dark period (averaged kinetics ±SD, n = 3).

Supplemental Figure S6

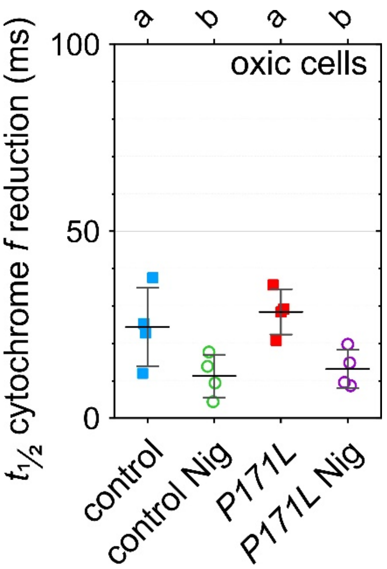

The cytochrome b_6f-related electron transfer restrictions in *PETC-P171L* cells is not established under aerobic conditions. The control and *PETC-P171L* cells displayed the same cytochrome-*f* reduction half-times upon a single cytochrome b_6f turnover under oxic conditions (means of n=4 ± SD are shown, minuscules on top indicate significances using One-Way ANOVA/Fisher-LSD, $P < 0.05$). The kinetics were accelerated upon nigericin (Nig) addition in both strains.

Supplemental Figure S7

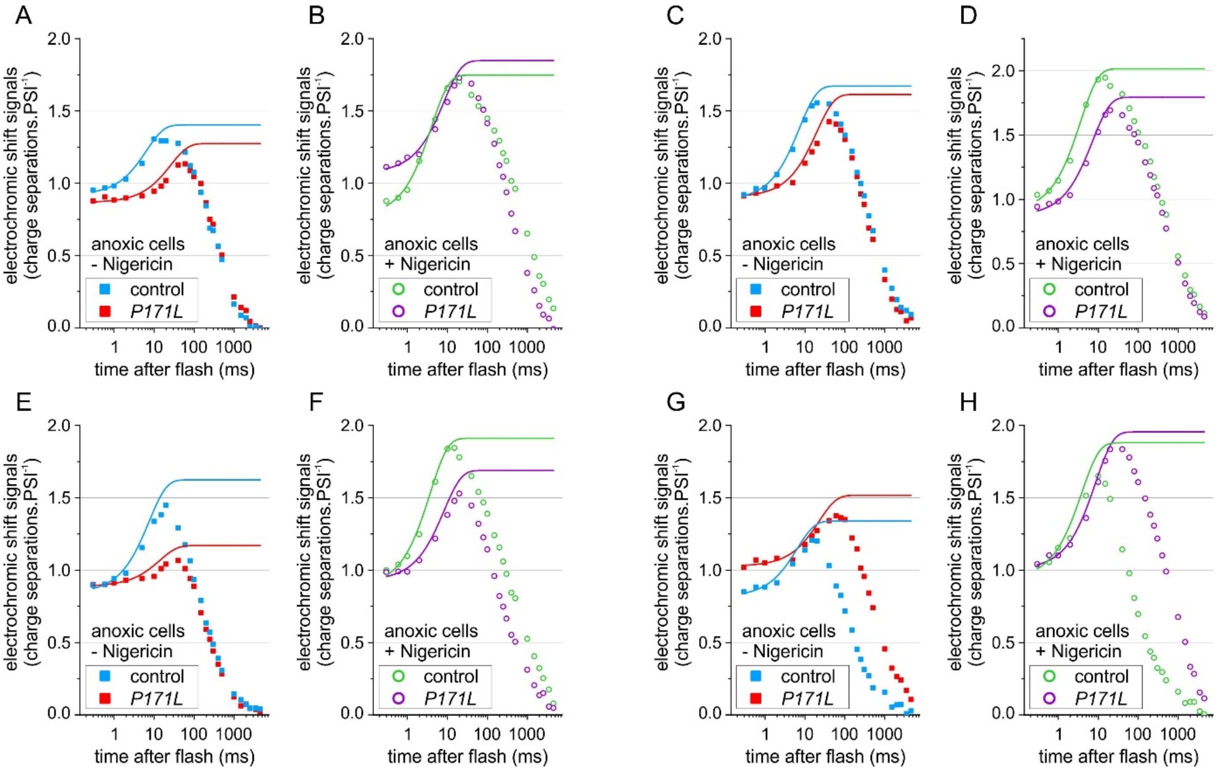

Anaerobic conditions interfere with the electrogenic activity of the *PETC-P171L* cytochrome b_6f complex which is sensitive to nigericin. Four biological replicates are paired (A-B, C-D, E-F, G-H) in the absence (A, C, E, G) and presence of nigericin (B, D, F, H). The associated decrease of the *PETC-P171L* electrogenic activity in the low-potential chain during the Q-cycle produced a less pronounced *b*-phase (solid line). Nigericin addition recovered a substantial *b*-phase in the *PETC-P171L* strain. The *b*-phase was quantified in Figure 4B of the main text.

Supplemental Figure S8

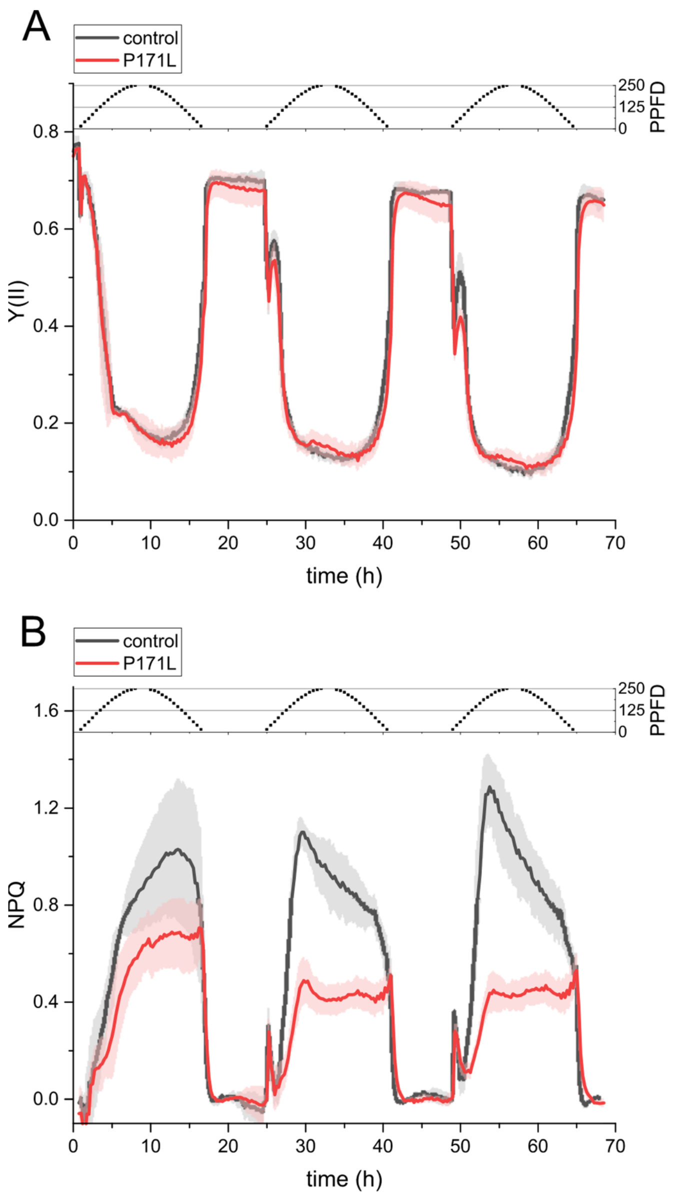

CO_2 limitation reveals the chlorophyll fluorescence phenotype of a limited photosynthetic capacity in *PETC-P171L* at lower irradiances.

Averaged photobioreactor experiments (n = 3 ± SD) of non-aerated cultures are shown for control and *PETC-P171L* cells. **(A)** The PSII quantum yield Y(II) and **(B)** NPQ show that *PETC-P171L* performance is less efficient in the twilight and throughout the day, respectively. The differences were amplified progressively during the three recorded photoperiods (16-h light / 8-h darkness). Photosynthetic Photon Flex Density (PPFD) is expressed as µmol photons m^{-2} s^{-1}.

Supplemental Figure S9

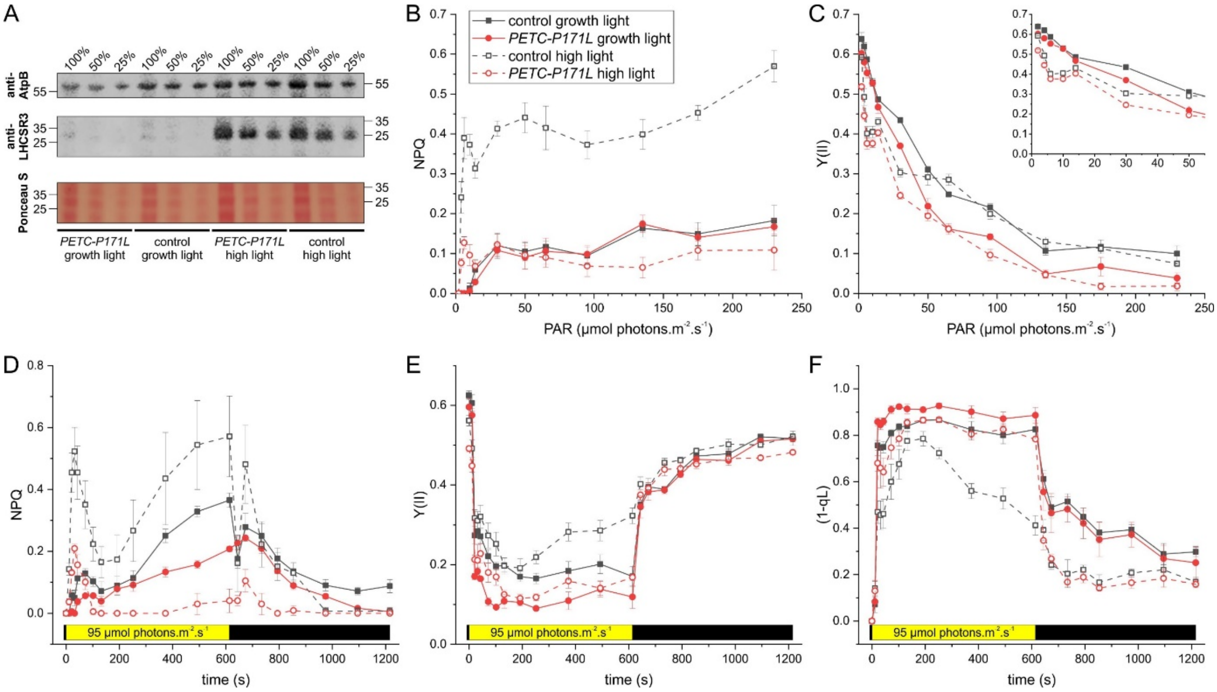

The chlorophyll fluorescence parameters reveal a limited photosynthetic capacity in *PETC-P171L*.

(A) Immunodetection of LHCSR3 protein was obtained from growth light samples (50 µmol photons·m^{-2}·s^{-1}) and after 4 h high light treatment (500 µmol photons·m^{-2}·s^{-1}). Based on chlorophyll loading (100% = 3 µg) and the AtpB reference, LHCSR3 accumulation and induction was comparable in the control and *PETC-P171L* cells. A representative Western Blot is shown and the chlorophyll fluorescence parameters in panels B to F are means three biological replicates (± standard deviations). **(B)** The control strains showed more NPQ during light curve experiments which was further increased upon high light treatment, unlike in *PETC-P171L*. **(C)** The PSII quantum yield, Y(II), was comparable in growth light samples up to 30 µmol photons·m^{-2}·s^{-1} of actinic light (inset), and Y(II) remained higher in the control strains when light was further increased. The strain-specific Y(II) developments showed a similar trend in high light samples. **(D)** The NPQ induction over 10 min of light was more efficient in control strains irrespectively of high light pre-treatment, although it enhanced NPQ capacity. **(E)** The corresponding Y(II) was also lower in *PETC-P171L* during the light period. The Y(II) in high light treated mutants increased rapidly upon transition to darkness and **(F)** the increased (1-qL) in those samples suggested a strongly reduced plastoquinone pool during the preceding illumination.

Supplemental Figure S10

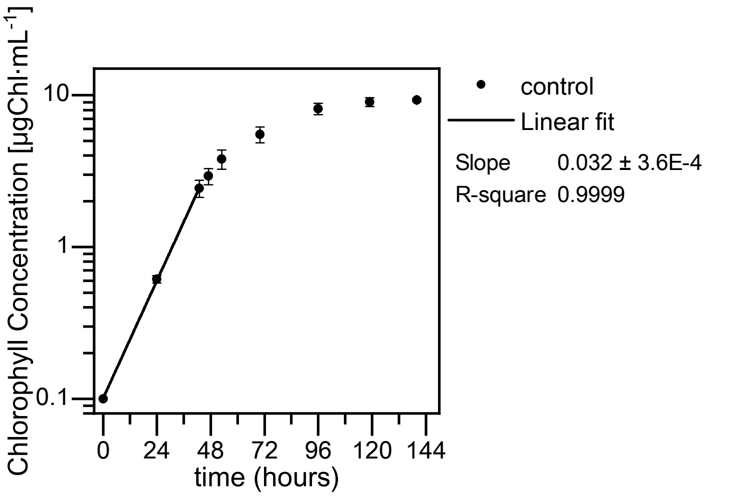

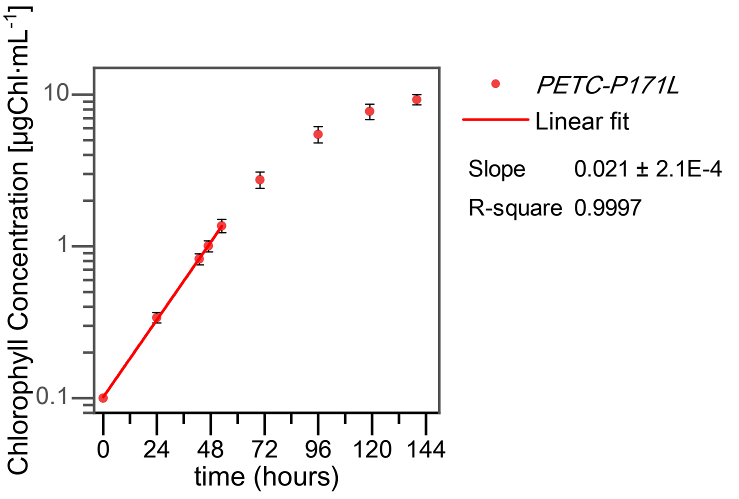

Linear approximation for logarithmic growth curve in common logarithmic axis.

The computed linear fitting (Linear fit) and the calculated slope against common logarithmic axis (Slope) and coefficient of determination (R-square) are shown (three biological replicates). The doubling times were calculated by dividing 0.30 by the value of Slope.

Supplemental Figure S11

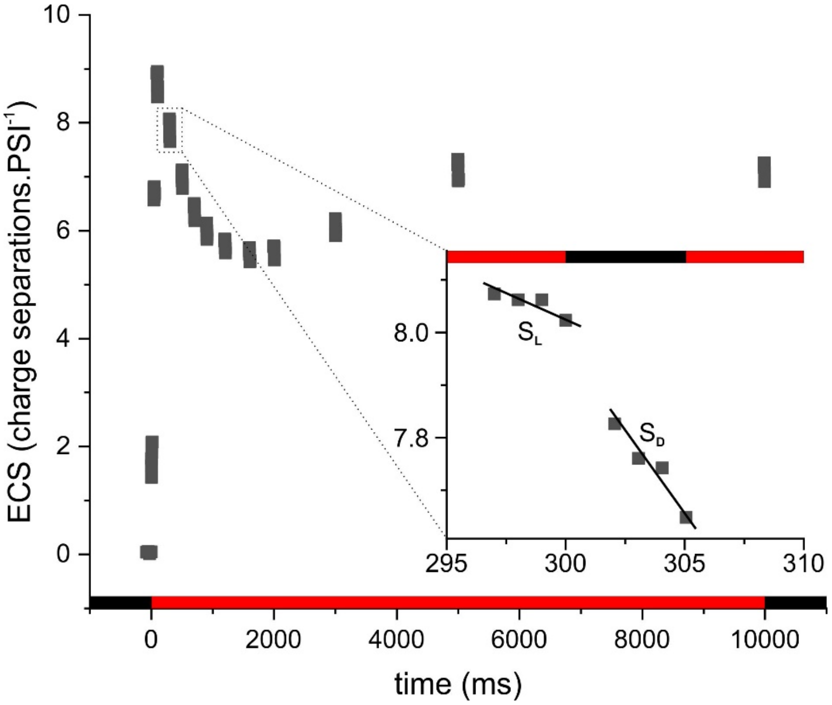

Dark Interval Relaxation Kinetics are shown in light-adapted cells. The samples experienced 30-s darkness before the 10-s continuous light (red bar). At distinct time points, the slope of electrochromic shift signals were recorded before shutting off light briefly (S_L), and during the 5-ms dark interval (S_D, magnified in the inset for the 300-ms illumination measurements). The photochemical rates shown in the main text (and expressed as charge separations·PSI^{-1}·s^{-1}) were calculated from S_L–S_D, and the protocol has been initially described as dark pulse method (Joliot and Joliot, 2002).

Supplemental Table S1

Primers

The primers used in this study were listed in the following. The mutations substituting 171st Pro to Leu in PETC (CCC to CTG) are shown in bold.

Name	sequence
HK#1483	5'- TTACACCTGAGCTCCACTATTTTATAGCAC -3'
HK#1484	5'- TGGCTGAGAGAGACAATCAACACGC -3'
HK#1485	5'- GGGCATATGGCCATGCTGTCCTCCCGCCGT -3'
HK#1486	5'- CCTCTAGAATTACGCCCACCAGGGCTCCAGGCCG -3'
HK#1491	5'- TGTATCAATATTGTTGCGTTCGGGCACTCG -3'
HK#1492	5'- TGATCCTCCTGTGGCTAATTGACCGTGG -3'
If-BbvCI_P171L_gcC_F	5'- TCAGTACAACGCTGAGGGCAAGGTGGTCCGTGGCCCCGCTC**TG**CTGGTGAGCAGCAGCA -3'
If-SnaBI_gcC_R	5'- CGCAGTGGGCCAGGGCCAGCGACTGCAAAAGGAGGGATAC -3'
If-paDcC-NdeI_F	5'- CTACTCACAACAAGCCCATA -3'
ccC_mP171L_R	5'- CAG**CA**GAGCGGGGCCACGGACCACCTTG -3'
ccC_mP171L_F	5'- GCCCCGCTC**TG**CTGTCGCTGGCCCTGGCCC -3'
If-paDcC-XbaI_R	5'- GTCCAGCTGCTGCCATCTAG -3'
cC_cdn131-137_F	5'- AACGCCGTGTGCACTCACCT -3'
cC_cdn137-131_R	5'- AGGTGAGTGCACACGGCGTT -3'

Supplemental Appendix S1

Formula for doubling time calculation with specific growth rate

By considering 1st order kinetics, the increment rate of chlorophyll concentration (*c*) against time (*t*) is described with specific growth rate *(k*) in the following equation.

$$\frac{dc}{dt} = kc$$

Deform this equation and we obtain the equation (1).

$$\frac{1}{n} dc = kdt \qquad (1)$$

Integrate from the initial point (0) to a point (a) about the equation (1), and we obtain the equation (2).

$$\int_0^a \frac{1}{c} dc = \int_0^a kdt$$

$$ln\, c_a - ln\, c_0 = kt_a - kt_0$$

$$ln \frac{c_a}{c_0} = k(t_{a-}t_0) \qquad (2)$$

Define *T* as the doubling time which is from t_2 to t_1, and meantime, the chlorophyll concentration becomes twice, therefore we obtain the following equations.

$$T = \left(t_{t_2} - t_{t_1}\right)$$

$$\frac{c_{t_2}}{c_{t_1}} = 2$$

Substitute them to the equation (2) and we obtain the equation (3) after deformation.

$$ln2 = kT$$

$$T = \frac{ln2}{k} \qquad (3)$$

When we define *K* as the slope (corresponds specific growth rate) obtained on the common logarithmic y-axis graph with linear fit, the *k* is converted in the following formula because base of *k* is Napier number.

$$k = \frac{K}{log_{10}e}$$

Substitute this formula to *k* in the equation (3) and we obtain the equation (4).

$$T = \frac{ln2 \cdot log_{10}e}{K} \qquad (4)$$

When we consider three significant digits, the doubling time approximates in the formula (5) to calculate doubling time from specific growth rate.

$$T = \frac{0.693\ \times\ 0.434}{K} = \frac{0.30}{K} \qquad (5)$$
